# Magnitude of intestinal parasitic infections and associated factors among food handlers working at Woldia University student’s cafeteria, Northeastern Ethiopia: an institution based cross-sectional study

**DOI:** 10.1186/s13104-019-4777-z

**Published:** 2019-11-08

**Authors:** Birhan Alemnew, Yalemzewud Belay, Asmamaw Demis

**Affiliations:** 1 Department of Medical Laboratory Sciences, College of Health Sciences, Woldia University, Woldia, Ethiopia; 2 Department of Public Health, College of Health Sciences, Woldia University, Woldia, Ethiopia; 3 Department of Nursing, College of Health Sciences, Woldia University, P.O.Box:400 Woldia, Ethiopia

**Keywords:** Intestinal parasites, Food handlers, Student cafeteria, Woldia University

## Abstract

**Objectives:**

The main aim of this study was to assess the Magnitude of intestinal parasitic infections and associated factors among food handlers working at Woldia University Student’s cafeteria, Northeastern Ethiopia. Institutional based cross-sectional study was conducted among 256 study participants in Woldia university student’s cafeteria, Northern Ethiopia. Systematic random sampling method was used to select the study participants. Data was collected using a standardized questionnaire by direct interviewing of study participants. Logistic regression was carried out to identify factors associated with intestinal parasitic infections.

**Results:**

A total of 256 food handlers were enrolled making the overall magnitude of the intestinal parasite which was stool specimens positive for different diagnostic stages of parasites was found to be 43 (16.8%). *Entamoeba histolytica/dispar* was the most prevalent parasites 14 (5.5%), followed by *Giardia lamblia* 10 (3.9%). Lack of food safety training (AOR = 6.58; 95% CI 2.46–17.62), no regular medical checkup (AOR = 2.41; 95% CI 1.47–4.24), no handwashing practice after toilet by soap (AOR = 3.24; 95% CI 1.28–8.19), no handwashing practice before eating by soap (AOR = 4.03; 95% CI 1.64–9.91) and haven’t food preparation license (AOR = 6.13; 95% CI 2.18–17.22) were significantly associated with parasitic infection among food handlers.

## Introduction

Intestinal parasitic infections are a major public health problem worldwide and assessed to infect about one-third of the total population; particularly in low and middle-income countries (LMIC) and constituting the greatest cause of illness and disease in children’s and food handlers with up to 2 million deaths are estimated per year [1–3]. According to WHO estimates, more than half a million deaths per year in Africa are due to food and water-borne related diseases which shows the tip of the iceberg, as many more cases that are sporadic went unreported [4]. In Ethiopia, intestinal parasitic infections are the major causes of mortality and morbidity causing a series of public health problems such as malnutrition, anaemia, and growth retardation as well as a higher susceptibility to other infections [1, 2]. Poor environmental sanitation, irrigation, overcrowding, resettlement, and low altitude were suggested to be responsible for the high prevalence of intestinal parasitic infection in the country [3–5]. Proper educations, protocols, training programs on the hygienic practices and regular medical checkup among food-handlers help to control the prevalence of intestinal pathogens and parasites at certain limits [2, 6].

Different studies conducted in Ethiopia showed that there was a high magnitude of enteric pathogens among food handlers working at university student’s cafeteria, due to improper hygienic practices, improper fingernail trimming and unavailability of standard protocols [2, 7, 8]. The most prevalent intestinal protozoan parasites in Ethiopia are *Giardia lamblia* and *Entamoeba histolytica/dispar*. Helminthic infection includes *Ascaris* lumbricoides, *Trichuris trichuriasis* and *Taenia* species [8, 9]. In spite of the public health importance of these intestinal parasites and the potential consequences, there was no enough data about the magnitude of intestinal parasites among food handlers in Woldia University. Therefore, this study was aimed to assess the magnitude and factors associated with intestinal parasitic infections among food handlers working at Woldia University Student’s cafeteria, Northeastern Ethiopia.

## Main text

### Methods

#### Study setting, design and period

Institutional based cross-sectional study design was conducted from March to June 2018 at Woldia University student cafeteria. Woldia University is one of the higher governmental university in Ethiopia which was found in Amhara region and located 521 km far from Addis Ababa, the capital city of Ethiopia. It has two campuses namely, Woldia (main campus) and Merssa College of Agriculture campus but our study was conducted in the main campus at Woldia.

### Sample size determination and sampling technique

The sample size was determined by a computer based on Epi info 7 software Stat Cal using single population proportion formula. The actual sample size is calculated using a single population proportion formula.

$${\text{n }} = \frac{{\left( {{\text{Z}}_{\alpha / 2} } \right)^{ 2} {\text{p }}\left( { 1- {\text{P}}} \right)}}{{{\text{d}}^{ 2} }} = \frac{{\left( { 1. 9 6} \right)^{ 2} *0. 5 2 3\left( { 1- 0. 5 2 3} \right)}}{{\left( { 0. 0 5} \right)^{ 2} }} = { 383} ,$$

where n is the minimum sample size required for the study; Z is the standard normal distribution (Z = 1.96) with a confidence interval of 95%; P is the prevalence of intestinal parasitic infections in Mekelle University, Ethiopia (p = 52.3%) [10]. d is a tolerable margin of error (d = 0.05)

Since the total number of source population was 595 and below 10,000 a correction formula was used to adjust the sample size as follows:

$${\text{nf}} = \frac{\text{n}}{{1 + {\text{n/N}}}} = \frac{383}{1 + 383/595} = 233.$$

By considering a 10% non-response rate, the final sample size of the study was 256.

### Method of data collection

Socio-demographic data and associated risk factors including institution and home-related data were collected using pre-tested structured questionnaire in a face to face interview. Stool specimens were collected from food handlers in a clean stool cup pre-labelled with study subject identification code at Woldia health center. A portion of the specimen (approximately 1–2 mg) was processed using direct saline preparation examined under 10× and 40× objective lenses of a light microscope for detection of helminths ova and larvae, protozoa cysts and trophozoites. After completion of direct stool examination, 1 g of each sample was emulsified in 10% formalin solution and examined in formol-ether concentration technique.

### Data quality assurance

The training was given for data collectors and supervisors about techniques of data collection and briefed on each question included in the data collection tool. The pre-test was conducted to ensure the validity of the tool, and then the correction was made before the actual data collection. Principal investigator and supervisors were checked on the spot and reviewed all the questionnaires to ensure completeness and consistency of the information collected and immediate action was taken accordingly. Double data entry was done by two data clerks and consistency of the entered data was cross-checked by comparing the two separately entered data.

### Data processing and analysis

The collected data were coded, cleaned, and entered into Epi data version 4.2 and exported to SPSS window version 24 for analysis. The binary logistic regression model was used to assess the association between independent variables and intestinal parasitic infections. All variables with P-value ≤ 0.25 in the bivariable analysis were included in the final model of multivariate analysis to control all possible confounders. Multi-collinearity was checked to see the linear correlation among the independent variables by using standard error. Variables with a standard error of > 2 were dropped from the multivariable analysis. Model fitness was checked with the Hosmer–Lemeshow test. Adjusted odds ratio with 95% CI was estimated to identify the factors associated with Intestinal parasitic infections using multivariable logistic regression analysis level of statistical significance was declared at p-value < 0.05.

## Results

### Socio-demographic characteristics and hygienic practice of food handlers

A total of 256 study participants were involved in the study with a response rate of 100%. Among them 164 (64.1%) food handlers were female. The mean age of the study participants was 25.24 (± 3.26 SD) years. About 116 (45.5%) were in age groups of 20–24 years. The majority of food handlers 210 (82%) had attended primary and above education. Of the total study participants, 205 (80.1%) had completely trimmed their nails, 230 (89.8%) were used hair cover during work, 209 (81.6%) of food handlers were used gown (Additional file 1: Table S1).

### Working House condition and practice-related factors of food handlers

Concerning working house condition, 105 (41%) and 106 (41.4%) had one room and window respectively. One hundred thirty-nine (54.3%) had fair ventilation system whereas 183 (71.5%) had good illumination systems. Regarding latrine condition, 149 (58.2%) had latrine with segregated water and 196 (76.6%) has clean by cleanliness (Table 1).

**Table 1:** Working house condition and practice of food handlers working at Woldia University Student’s cafeteria, Northeastern Ethiopia, 2018 (N = 256)

Variables	Characteristics	Frequency (n = 256)	Percentage
Houseroom of food handlers	One room	105	41.0
	Two rooms	69	27.0
	Three rooms	82	32.0
Number of windows for food handlers	One window	106	41.4
	Two windows	78	30.5
	Three windows	72	28.1
Ventilation system of the house for food handlers	Good	90	35.2
	Fair	139	54.3
	Bad	27	10.5
Illumination system of the house for food handlers	Good	183	71.5
	Bad	73	28.5
Type of floor of the house for food handlers	Cement	137	53.5
	Soil	119	46.5
Type of kitchen the house of food handlers	Attached to the main house	87	34.0
	Detached to the main house	169	66.0
Cleanliness of the house of food handlers	Very good	97	37.9
	Good	159	62.1
Use of water of food handlers	Private pipe	110	43.0
	Common pipe	146	57.0
Type of latrine for food handlers	Has water segregation	149	58.2
	No water segregation	107	41.8
Cleanliness of latrine for food handlers	Clean	196	76.6
	Not clean	60	23.4
Liquid disposal methods for food handlers	Open field	90	35.2
	Use segregation	166	64.8
Solid waste disposal system of food handlers	Hole	170	66.4
	Open field	49	19.1
	Municipal body	37	14.5
Use of hair cover during food preparations	Yes	230	89.8
	No	26	10.2

### The magnitude of intestinal parasite

Both direct microscopic and concentration techniques were used for identifying intestinal parasites from 256 stool specimens of food handlers. The overall prevalence of intestinal parasite positive for different intestinal parasite species was found to be 16.8% (95% CI 12.1%–21.5%). *Entamoeba histolytica/dispar* was the most prevalent parasites 14 (5.5%), followed by *Giardia lamblia* 10 (3.9%), *Ascaris lumbricoides* 9 (3.5%), *Strongloid stercolaris* 3 (1.2%), *Trichuris trichiura* 3 (1.2%), *Taenia* species 2 (0.8%), and *Hookworm* 2 (0.8%).

### Factors associated with intestinal parasitic infection

The odds of food handlers who didn’t always wash their hand after toilet by soap were three times more likely acquired intestinal parasitic infection than those who wash their hands after toilet by soap (AOR = 3.24; 95% CI 1.28–8.19). Concerning food handlers regular medical checkup and had food preparation license, food handlers who had regular medical checkup were 2.4 times (AOR = 2.41; 95% CI 1.47–4.24) and food handlers who had food preparation license were 6.13 times (AOR = 6.13; 95% CI 2.18–17.22) more likely acquired intestinal parasitic infection as compared with their counterparts. We also found that the odds of having food safety hygiene and training practices were 6.58 times higher (AOR = 6.58; 95% CI 2.46–17.62) among food handlers than food handlers that had no training opportunity. Those food handlers who did not trim their fingernail were 3.11 times more likely (AOR = 3.11; 95% CI 1.14–8.67) infested than those who did regularly (Table 2).

**Table 2:** Bivariable and multivariable logistic regression analysis of factors associated with intestinal parasitic infections among food handlers working at Woldia University Student’s Cafeteria, Northeastern Ethiopia, 2019 (n = 256)

Variables	Intestinal parasitic infections		COR (95% CI)	AOR (95% CI)
	Positive (%)	Negative (%)		
Sex				
Male	15 (16.3)	77 (83.7)	1	1
Female	28 (17.1)	136 (82.9)	0.95 (0.48–1.88)	1.79 (0.62–5.18)
Age				
15–19	4 (30.8)	9 (69.2)	0.41 (0.11–1.47)	1.91 (0.40–9.09)
20–24	18 (15.5)	98 (84.5)	1.00	1.00
25–29	12 (16.9)	59 (83.1)	0.89 (0.40–1.99)	0.53 (0.06–5.10)
30–34	5 (17.2)	24– (82.8)	0.84 (0.28–3.49)	1.31 (0.27–6.41)
≥ 35	4 (14.8)	23 (85.2)	1.04 (0.32–3.38)	1.71 (0.24–12.22)
Hand washing after toilet with a soap				
Yes	14 (7.6)	169 (82.4)	1	1
No	29 (39.7)	44 (60.3)	7.96 (3.87–16.32)	3.24 (1.28–8.19)*
Hand washing before eating food with a soap				
Yes	17 (8.6)	182 (81.4)	1	1
No	26 (45.6)	31 (54.4)	8.98 (4.37–18.45)	4.03 (1.64–9.91)**
Fingernail trimming				
Yes	22 (10.7)	183 (89.3)	1	1
No	21 (41.2)	30 (58.8)	3.76 (1.88–7.51)	3.11 (1.14–8.67)***
Use of apron when handling food				
Yes	37 (17.7)	172 (82.3)	1	1
No	6 (12.8)	41 (87.2)	0.68 (0.27–1.72)	0.37 (0.09–1.50)
Use of hair cover during work of food handlers				
Yes	38 (16.5)	192 (83.5)	1	1
No	5 (19.2)	21 (80.8)	1.20 (0.43–3.34)	0.70 (0.17–2.81)
Year of service				
< 1 year	9 (18.7)	39 (81.3)	0.90 (0.40–2.03)	0.29 (0.05–1.71)
1–5 years	32 (17.6)	150 (82.4)	1	1
6–10 years	2 (8.3)	24 (81.7)	2.54 (0.57–11.31)	0.54 (0.08–3.84)
Food safety training				
Yes	22 (10.6)	186 (89.4)	1	1
No	21 (43.7)	27 (52.3)	6.57 (3.20–13.52)	6.58 (2.46–17.62)****
Medical checkup for IPs				
Yes	25 (11.8)	186 (88.2)	1	1
No	18 (40.0)	27 (60.0)	3.06 (1.45–6.47)	2.41 (1.47–4.24)***
Having a license for food handling practice				
Yes	23 (11.1)	184 (88.9)	1	1
No	20 (40.8)	29 (59.2)	5.52 (2.70–11.20)	6.13 (2.18–17.22)****
Took medication				
Yes	16 (27.6)	42 (72.4)	1	1
No	27 (13.6)	171 (86.4)	2.41 (1.19–4.89)	0.51 (0.18–1.39)

Significant at: *P = 0.013, **P = 002, ***P = 0.03, ****P = 0.001, 1 = reference

## Discussion

The overall prevalence of intestinal parasitic infection among food handlers working in Woldia university student cafeteria was 16.8%. This finding was similar with studies from different parts of Ethiopia such as 14.5% Aksum Town [13], 15% Wollo University student’s cafeteria [14]. However, the prevalence of IPIs was low as compared to studies conducted in Ethiopia, 41.1% in Bahir Dar, Amhara [15], 52.4% Mekelle University student cafeteria, Tigray [10], 45.3% Addis Ababa University student’s cafeteria, Addis Ababa [11], 33%, in Jimma University Specialized Hospital, Southwest Ethiopia [16], 36% Arba Minch University, Southern Ethiopia [19], 25.2% Haramaya University cafeterias, Eastern Ethiopia [12], 61.9% among food handlers at Prison, East and West Gojjam [5] and 43.38% Saudi Arabia [18]. The possible reason might be due to good personal hygiene practices and environmental sanitation, continuous health-promotion practices, time of the study and geographical variation of the participants.

In our study*, Entamoeba histolytica/dispar* was the most prevalent parasites 14 (5.5%), followed by *Giardia lamblia* 10 (3.9%) among food handlers working in Woldia university student cafeteria. In contrast, the most abundant parasite of Arba Minch University students’ cafeteria, were *Entamoeba histolytica/dispar* 48 (14%) followed by *Ascaris lumbricoides* 32 (9.27%) [17], Haramaya University cafeterias were *Entamoeba histolytica/dispar* (46.7%) and *Ascaris lumbricoides* (14.3%) [12], Wollo University student’s cafeteria were *Enttamoeba. histolytica* (5.5%) followed by *Ascaris lumbricoides* (4%) and then *Gardia lamblia* (3%) [14]. However, the predominant organism were found to be *Ascaris lumbricoides* in other studies which was conducted in Jimma University Specialized Hospital [16] and Gondar Ethiopia [9]. This might be due to food habits, cultural factors and geographical conditions.

These findings indicated that lack of food safety training, no regular medical checkup, no handwashing practice after toilet and after eating by soap were significantly associated with parasitic infection among food handlers. The odds of food handlers who didn’t always wash their hand after toilet by soap were three times more likely acquired intestinal parasitic infection than those who wash their hands after toilet, which is supported with the study conducted in Arba Minch University, Ethiopia [19], Haramaya University [12] and at Prison, East and West Gojjam [5]. This might be due to the fact that those food handlers who didn’t wash their hands with soap after toilet may infest with intestinal infection during eating, drinking and cooking.

The odds of food handlers who hadn’t received regular a medical checkup were 2.4 times more likely infested with intestinal parasites as compared with those food handlers who had received medical checkup. This is in line with the study conducted in Aksum Town [13] and Wollo University student’s cafeteria [14]. This might be due to the fact that those food handlers who hadn’t received medical checkup may not know their status, as a result, they became a carrier and infested with intestinal parasites.

Food handlers who hadn’t taken food safety training were 6.58 times more infested with intestinal parasites as compared with food handlers who took food safety training. This is in line with the study conducted in Aksum Town [13] and Dessie town [6]. This might be due to the fact that those food handlers who didn’t take food safety training may not understand the way of prevention and control mechanism, as a result, they became highly infested with intestinal parasites.

Food handlers who did not trim their fingernail were 3 times more likely infested with intestinal parasites than those who trimmed their fingers regularly. This is similar to the study conducted in Wollo University student’s cafeteria [14], Arba Minch University, Ethiopia [19] and at Prison of East and West Gojjam [5]. This might be due to the fact that those food handlers didn’t trim their fingernail which makes them carrier of parasites in the fingernail, as a result, they became more infested with intestinal parasites.

## Conclusions

Generally, the overall magnitude of intestinal parasitic infection among food handlers at Woldia university student cafeteria was found relatively low as compared from another public university within the country. However, food safety training, fingernail trimming practice, handwashing with soap after toilet, hand washing after eating and medical checkups were the potential factors for intestinal parasitic infection. Therefore, regular and contentious training on food safety and handwashing practice and regular periodic medical checkup are essential strategies for prevention of intestinal parasitic infections. Besides, further studies should be undertaken on bacterial isolates and associated risk factors among those food handlers at the study area.

## Limitations of the study

The cross-sectional nature of the study design limits the applicability of the findings in establishing causality between the variables.

## Supplementary information

**Additional file 1: Table S1.** Distribution of socio-demographic characteristics and hygienic practice of food handlers working at Woldia University Student’s cafeteria, Northeastern Ethiopia, 2018 (N=256).

## Data Availability

All related data has been presented within the manuscript. The dataset supporting the conclusions of this article is fully available from the authors on request.

## Abbrevations

AOR: Adjusted Odds Ratio
CDC: Center for Disease Control and Prevention
CI: Confidence Interval
IPIs: Intestinal Parasitic Infections
IRB: Institutional Review Board
SPSS: Statistical Package for Social Sciences
WDU: Woldia University
WHO: World Health Organizations
