# Supplementary material for: Magnitude of intestinal parasitic infections and associated factors among food handlers working at Woldia University student’s cafeteria, Northeastern Ethiopia: an institution based cross-sectional study
Source: BMC Res Notes. 2019 Nov 8;12:736. doi: 10.1186/s13104-019-4777-z (PMC6844021; doi:10.1186/s13104-019-4777-z)
Supplement: Supplementary file 1 — Additional file 1: Table S1. Distribution of socio-demographic characteristics and hygienic practice of food handlers working at Woldia University Student’s cafeteria, Northeastern Ethiopia, 2018 (N=256). [file 13104_2019_4777_MOESM1_ESM.docx]

Table S1: Distribution of socio-demographic characteristics and hygienic practice of food handlers working at Woldia University Student’s cafeteria, Northeastern Ethiopia, 2018, (N=256).

| Variables | Characteristics | Frequency | Percentage (%) |
| --- | --- | --- | --- |
| Sex | Male | 92 | 35.9 |
|  | Female | 164 | 64.1 |
| Age in years | 15-19 | 13 | 5.1 |
|  | 20-24 | 116 | 45.3 |
|  | 25-29 | 71 | 27.7 |
|  | 30-34 | 29 | 11.3 |
|  | ≥ 35 | 27 | 10.5 |
| Educational status | Illiterate | 14 | 5.5 |
|  | Read and write | 32 | 12.5 |
|  | Primary education | 156 | 60.9 |
|  | Secondary & above | 54 | 21.1 |
| Marital status | Married | 88 | 34.4 |
|  | Single | 129 | 50.4 |
|  | Divorced | 25 | 9.8 |
|  | Widowed | 14 | 5.5 |
| Religion | Orthodox | 218 | 85.2 |
|  | Muslim | 38 | 14.8 |
| Use of hand washing after toilet | Soap and water | 183 | 71.5 |
|  | Water | 73 | 28.5 |
| Use of hand washing before touching foods of food handlers | Soap and water | 199 | 77.7 |
|  | Water | 57 | 22.3 |
| Nail trimming | Yes | 205 | 80.1 |
|  | No | 51 | 19.9 |
| Use of gown during work of food handlers | Yes | 209 | 81.6 |
|  | No | 47 | 18.4 |
| Use of hair cover during work of food handlers | Yes | 230 | 89.8 |
|  | No | 26 | 10.2 |
| Type of your responsibility in cafeteria of food handlers | Food coking | 100 | 39.1 |
|  | Waiter | 116 | 45.3 |
|  | Collect utensil materials | 40 | 15.6 |
| Your service of food handlers | Less than one year | 48 | 18.8 |
|  | 1-5 years | 182 | 71.1 |
|  | 6-10 years | 26 | 10.2 |
| Have you trained about food handling | Yes | 208 | 81.3 |
|  | No | 48 | 18.8 |
| Medical check-up of food handlers | Yes | 211 | 82.4 |
|  | No | 45 | 17.6 |
| Have you license for food handling practice | Yes | 207 | 80.9 |
|  | No | 49 | 19.1 |
| Had you took medication for intestinal parasite & bacteria before now | Yes | 58 | 22.7 |
|  | No | 198 | 77.3 |
